# Integrated Sugar–Acid–Phenolic Profiling Identifies Distinct Chemical Types Associated with Antioxidant Capacity in Apple Pulp Samples

**DOI:** 10.3390/foods15183353

**Published:** 2026-09-21

**Authors:** Haorui Zheng, Hengyang Zhang, Taoyu Wang, Tong Peng, Lisha Zhong, Keke Fu, Yang Cao, Xiaoyu Zhang, Lin Tang

**Affiliations:** 1 College of Life Sciences, Sichuan University, Chengdu 610000, China; 2 Department of R&D, Keystonecare Technology (Chengdu) Co., Ltd., No. 200 Tianfu 5th Street, Chengdu 610094, China; 3 College of Life Sciences, Sichuan Normal University, Chengdu 610101, China

**Keywords:** apple pulp, sugar, organic acids, phenolic compounds, antioxidant activity, HepG2 cells, chemical classification

## Abstract

This study characterized the sugar, organic acid, and phenolic profiles of 25 apple pulp samples from 12 cultivars to investigate their associations with antioxidant potential. Fructose and malic acid were the predominant free sugar and organic acid, respectively. Hierarchical clustering based on seven non-redundant chemical traits classified the samples into four distinct chemical types: acid-rich, sugar-enriched/low-phenolic, intermediate/low-accumulation, and phenolic-rich types. Phenolic-related traits showed stronger associations with in vitro antioxidant capacity than sugar- and acid-related traits. For cellular validation, polyphenol extracts from five representative samples were tested at 5 μg/mL, a concentration selected based on cell viability results under the tested conditions. The extracts significantly reduced H_2_O_2_- and *t*-BHP-induced intracellular ROS accumulation in HepG2 cells, with the TPGSH sample exhibiting the strongest cellular protection. These findings establish an integrated chemical–antioxidant framework for characterizing apple pulp samples and identifying compositional patterns associated with antioxidant activity.

## 1. Introduction

Apple (*Malus* × *domestica* Borkh.) is one of the most widely produced and traded temperate fruits worldwide. According to FAOSTAT, global apple production reached approximately 97 million tonnes in 2023, with China contributing about half of the total output; Turkey, the United States, Poland, and India were also major producers [1]. International trade is similarly substantial, with Italy, China, the United States, New Zealand, and Chile among the leading international exporters of fresh apples by export value in 2023 [2]. The nutritional and functional value of apple pulp is closely related to its chemical composition. Soluble sugars and organic acids are important primary metabolites, whereas phenolic compounds, including flavan-3-ols, procyanidins, phenolic acids, and flavonols, are important secondary metabolites associated with antioxidant potential [3,4,5].

Previous studies have separately investigated the sugars, organic acids, phenolic compounds, or antioxidant capacities of apples [6,7,8,9,10,11]. However, it remains unclear how different chemical dimensions, particularly primary metabolites, such as sugars and organic acids, and secondary metabolites, such as phenolic compounds, contribute to variation in the functional properties of apple pulp. The relationships among individual chemical classes and antioxidant activities cannot be adequately resolved by analyzing a single chemical group or antioxidant assay in isolation. Moreover, cell-free assays, such as ABTS, DPPH, and FRAP, reflect different chemical reaction mechanisms and do not fully account for cellular uptake, metabolism, or endogenous antioxidant responses. An integrated analysis, combining chemical profiling, complementary antioxidant assays, and cellular oxidative stress models, is therefore required to clarify the associations between apple pulp composition and antioxidant potential.

Accordingly, this study analyzed 25 apple pulp samples from 12 cultivars obtained from multiple apple-producing regions in China and selected foreign origins. Free sugars, organic acids, total phenols, total flavonoids, and representative phenolic and flavonoid compounds were quantified, while additional phenolic compounds were putatively annotated using non-targeted mass spectrometry. Seven non-redundant chemical traits were used for chemical classification, and the resulting chemical types were evaluated in relation to ABTS, DPPH, and FRAP antioxidant capacities. In addition, polyphenol extracts from five representative samples were tested in H_2_O_2_- and *t*-BHP-induced oxidative stress models in HepG2 cells. We hypothesized that phenolic-related traits would show stronger associations with antioxidant capacity than sugar- and acid-related traits. This study aimed to establish an integrated chemical–antioxidant framework for interpreting sample-level differences in the functional properties of apple pulp.

## 2. Materials and Methods

### 2.1. Plant Materials

Twenty-five apple pulp samples from 12 cultivars were collected from major apple-producing regions in China, including Sichuan, Shaanxi, Shanxi, Gansu, Jilin, Shandong, Xinjiang, and Yunnan, as well as selected foreign ori-gins, including Australia, the United States, and New Zealand (Table S1). Sampling was conducted according to the National Standard of China for Fresh Apples [12]. For each sample, 100 commercially mature “Extra Class” fruits without visible mechanical damage, disease symptoms, or physiological disorders were randomly selected. Fruit maturity was assessed according to the commercial maturity requirements and available physicochemical maturity indicators. Fruits were harvested by hand when applicable and transported at 0 ± 0.5 °C. All samples were deliv-ered to the laboratory within 48 h.

For each apple sample, the 100 fruits were randomly allocated into five groups, with 20 fruits in each group. One fruit was randomly selected from each group and analyzed independently, resulting in five individual-fruit biological replicates per sample (*n* = 5). The pulp from each selected fruit was separately homogenized within 24 h after arrival, aliquoted, and stored at −80 °C until analysis. Because the samples were obtained from commercial orchards or retail sources, harvest date, maturity stage, agronomic management, storage, and postharvest handling could not be completely standardized. Therefore, the observed compositional differences may reflect the combined effects of cultivar background, geographic origin, maturity status, orchard management, and postharvest conditions.

### 2.2. Extraction of Sugars, Organic Acids, and Phenolic and Flavonoid Compounds

For free sugar and organic acid analysis, 5 g of apple pulp was homogenized with 10 mL of deionized water containing 0.01% ascorbic acid. Ascorbic acid was used solely as an extraction stabilizer and was not quantified as an analyte. After centrifugation, the supernatant was collected. For free sugar analysis, the supernatant was mixed with an equal volume of chromatographic-grade acetonitrile. For organic acid analysis, the supernatant was directly filtered through a 0.22 μm membrane before instrumental analysis.

For the extraction of phenolic and flavonoid compounds, 10 g of apple pulp was extracted with 30 mL of 70% ethanol using ultrasonic-assisted extraction at 40 kHz and 40 °C for 30 min. The extraction was repeated twice, and the combined supernatants were concentrated by rotary evaporation. The residue was reconstituted in 10 mL of 70% methanol, filtered through a 0.22 μm membrane, and used for phenolic and flavonoid compound analysis and antioxidant assays. Extraction and sample handling were conducted under subdued light. Extracts were kept on ice and protected from light, where appropriate, to minimize oxidation.

### 2.3. Determination of Total Phenols and Total Flavonoids

Total phenolic and total flavonoid contents were determined using commercial assay kits according to the manufacturers’ instructions. The total phenolic content assay was based on the Folin–Ciocalteu colorimetric reaction, whereas the total flavonoid content assay was based on aluminum chloride complexation. The manufacturer, catalog number, reaction conditions, detection wavelength, and calibration range for each kit are provided in Supplementary Materials S1 and S2.

Total phenolic content was calculated from a gallic acid calibration curve and expressed as milligrams of gallic acid equivalents per 100 g fresh weight (mg GAE/100 g FW). Total flavonoid content was calculated from a rutin calibration curve and expressed as milligrams of rutin equivalents per 100 g fresh weight (mg RE/100 g FW). All measurements were performed using five biological replicates per sample.

### 2.4. Non-Targeted Profiling of Phenolic and Flavonoid Compounds

Non-targeted profiling of phenolic and flavonoid compounds was performed using an ExionLC AD system coupled with an X500R QTOF mass spectrometer (SCIEX, Framingham, MA, USA) operated in negative-ion mode. Data were acquired in information-dependent acquisition mode. Raw data were processed using SCIEX OS (SCIEX, Framingham, MA, USA) and MS-DIAL (RIKEN, Wako, Japan) for peak extraction, denoising, alignment, and annotation. Putative compound annotation was performed by matching accurate mass, isotopic patterns, and MS/MS fragmentation spectra against spectral databases. Candidate compounds with matching scores greater than 80% were retained. Compounds annotated without confirmation using authentic standards were reported as MSI Level 2 annotations [13]. Detailed chromatographic, mass spectrometric, and data-processing parameters are provided in Supplementary Material S2.

### 2.5. Targeted Quantification of Free Sugars and Organic Acids

Free sugars were analyzed by UPLC-ELSD (Waters Corporation, Milford, MA, USA) on an ACQUITY UPLC BEH Amide column (2.1 × 100 mm, 1.7 μm; Waters Corporation, Milford, MA, USA). The mobile phases consisted of acetonitrile containing 0.2% triethylamine and 50% aqueous acetonitrile containing 0.2% triethylamine. Glucose, fructose, and sucrose were quantified using external calibration curves prepared from authenticated standards.

Organic acids were analyzed by UPLC-PDA-MS (Waters Corporation, Milford, MA, USA) on an Atlantis Premier BEH C18 AX column (2.1 × 100 mm, 1.7 μm; Waters Corporation, Milford, MA, USA). Acetonitrile and an ammonium formate solution containing formic acid were used as mobile phases. Malic acid and quinic acid were quantified using authenticated standards. Mass spectrometric detection was performed in negative ion mode.

Relative sweetness was calculated using the sweetness coefficients of the quantified sugars: Relative sweetness = fructose × 1.7 + glucose × 0.7 + sucrose × 1.0 [14].

Total sugars were calculated as the sum of fructose, glucose, and sucrose. Total acids were calculated as the sum of quantified organic acids. The sugar/acid ratio was calculated as total sugars divided by total acids.

### 2.6. Targeted Quantification of Phenolic and Flavonoid Compounds

Targeted analysis of phenolic and flavonoid compounds was performed by UPLC-PDA-MS on a Cortecs Premier T3 column (2.1 × 150 mm, 1.6 μm; Waters Corporation, Milford, MA, USA). Chromatographic-grade acetonitrile and ammonium formate solution containing formic acid were used as mobile phases. Phenolic and flavonoid compounds were quantified using authenticated standards and external calibration curves. Sixteen representative phenolic and flavonoid compounds were quantified based on their known relevance in apple fruit and the results of non-targeted screening.

### 2.7. In Vitro Antioxidant Assays

The antioxidant capacities of apple polyphenol extracts were evaluated using three commercial assay kits according to the manufacturers’ instructions.

For the ABTS radical scavenging assay, the ABTS Antioxidant Capacity Assay Kit (Sangon Biotech Co., Ltd., Shanghai, China) was used. In this assay, the pre-formed ABTS radical cation (ABTS•^+^) is reduced by antioxidants, leading to a decrease in absorbance. The ABTS radical scavenging activity was determined by measuring the reduction in ABTS•^+^ at the characteristic absorption wavelength, using rutin as the standard for calibration. Results were expressed as the ABTS free radical scavenging rate (%).

For the DPPH radical scavenging assay, the DPPH Antioxidant Capacity Assay Kit (Sangon Biotech Co., Ltd., Shanghai, China) was used. DPPH is a stable free radical that can accept hydrogen atoms or electrons from antioxidants, resulting in a decrease in its characteristic absorbance. Therefore, the DPPH radical scavenging activity was evaluated by monitoring the loss of absorbance of the DPPH radical, with Trolox used as the standard for calibration. Results were expressed as the DPPH free radical scavenging rate (%).

For the FRAP reducing power assay, the FRAP Total Reducing Power Assay Kit (Beyotime Biotechnology Co., Ltd., Shanghai, China) was used. In this assay, antioxidants reduce the ferric-tripyridyltriazine (Fe^3+^-TPTZ) complex to the ferrous form (Fe^2+^-TPTZ), accompanied by an increase in absorbance. The reducing power of the samples was therefore determined by measuring the formation of Fe^2+^-TPTZ, using Trolox as the standard for calibration. Results were expressed as total reducing power (%).

All assays were performed using five biological replicates. For integrated comparison, ABTS, DPPH, and FRAP percentage values were independently normalized across the 25 apple pulp samples. A composite antioxidant score was calculated as the mean of the normalized values for each sample.

### 2.8. Cell Culture and Cytotoxicity Assessment

HepG2 cells were obtained from the Shanghai Institute of Cell Biology, Chinese Academy of Sciences (Shanghai, China). The cells were maintained in high-glucose Dulbecco’s Modified Eagle Medium (DMEM) supplemented with 10% fetal bovine serum (FBS) and 1% penicillin–streptomycin at 37 °C in a humidified incubator with 5% CO_2_. Based on chemical composition, in vitro antioxidant capacity, and chemical type representation, five apple pulp samples, AZ, XC, LF, TPGSH, and AKS, were selected for cellular antioxidant validation.

Cell viability was determined using the CCK-8 assay. HepG2 cells were treated with apple polyphenol extracts at different concentrations to evaluate potential cytotoxicity. Based on the viability results, 5 μg/mL was selected for subsequent cellular antioxidant experiments because it did not significantly reduce cell viability.

### 2.9. Establishment of Oxidative Stress Models and Cellular Antioxidant Assays

To establish oxidative stress models, HepG2 cells were exposed to different concentrations of H_2_O_2_ or *t*-BHP for different durations. Model conditions were selected based on an approximately 50% reduction in cell viability. The final oxidative injury conditions were 500 μM H_2_O_2_ for 1 h and 1 mM *t*-BHP for 1.5 h.

For cellular antioxidant assays, HepG2 cells were pretreated with apple polyphenol extracts or vitamin C as a positive control, followed by H_2_O_2_ or *t*-BHP exposure. Intracellular ROS levels were detected using the DCFH-DA fluorescent probe. Fluorescence intensity was measured at excitation/emission wavelengths of 488/525 nm. LDH release, MDA content, and the activities of SOD and CAT were determined using commercial assay kits according to the manufacturers’ instructions.

### 2.10. Chemical Typing and Correlation Analysis

To classify the apple pulp samples, seven non-redundant chemical traits, including fructose, glucose, sucrose, malic acid, quinic acid, total phenols, and total flavonoids, were standardized using Z-scores across the 25 samples. Total sugars was excluded because it was calculated from fructose, glucose, and sucrose, which could have disproportionately weighted sugar-related variation. Hierarchical clustering was performed using Euclidean distance and Ward.D2 linkage, and the dendrogram was divided into four sample-level chemical types. The resulting type labels were used only as cluster identifiers and did not represent a ranking of sample quality. Fruit photographs were used only for morphological reference and were not included as variables in the chemical clustering.

Spearman’s rank correlation coefficients were calculated between antioxidant indices, including ABTS, DPPH, FRAP, and the composite antioxidant score, and selected chemical traits.

### 2.11. Statistical Analysis

Data are expressed as the mean ± standard deviation of five biological replicates. For sample-level correlation analyses, the mean value of the five biological replicates was used for each apple pulp sample. Statistical analyses were performed using R version 4.5.2 and GraphPad Prism GraphPad Prism version 9.0.0 (GraphPad Software, San Diego, CA, USA).

One-way analysis of variance, followed by Tukey’s post hoc test, was used for comparisons among multiple samples where appropriate. Kruskal–Wallis tests were used to compare antioxidant scores among the four chemical types. Spearman’s rank correlation analysis was used to assess the associations between antioxidant indices and chemical traits. Statistical significance was defined as *p* < 0.05.

Figures were generated using the R packages ggplot2 (version 3.4.2), circlize (version 0.4.15), ComplexHeatmap (version 2.16.0), pheatmap (version 1.0.12), and patchwork (version 1.1.2), together with GraphPad Prism (version 9.0.0) where appropriate.

## 3. Results

### 3.1. Variations in Phenolic, Sugar and Organic Acid Composition Among Apple Pulp Samples

**Figure 1:**
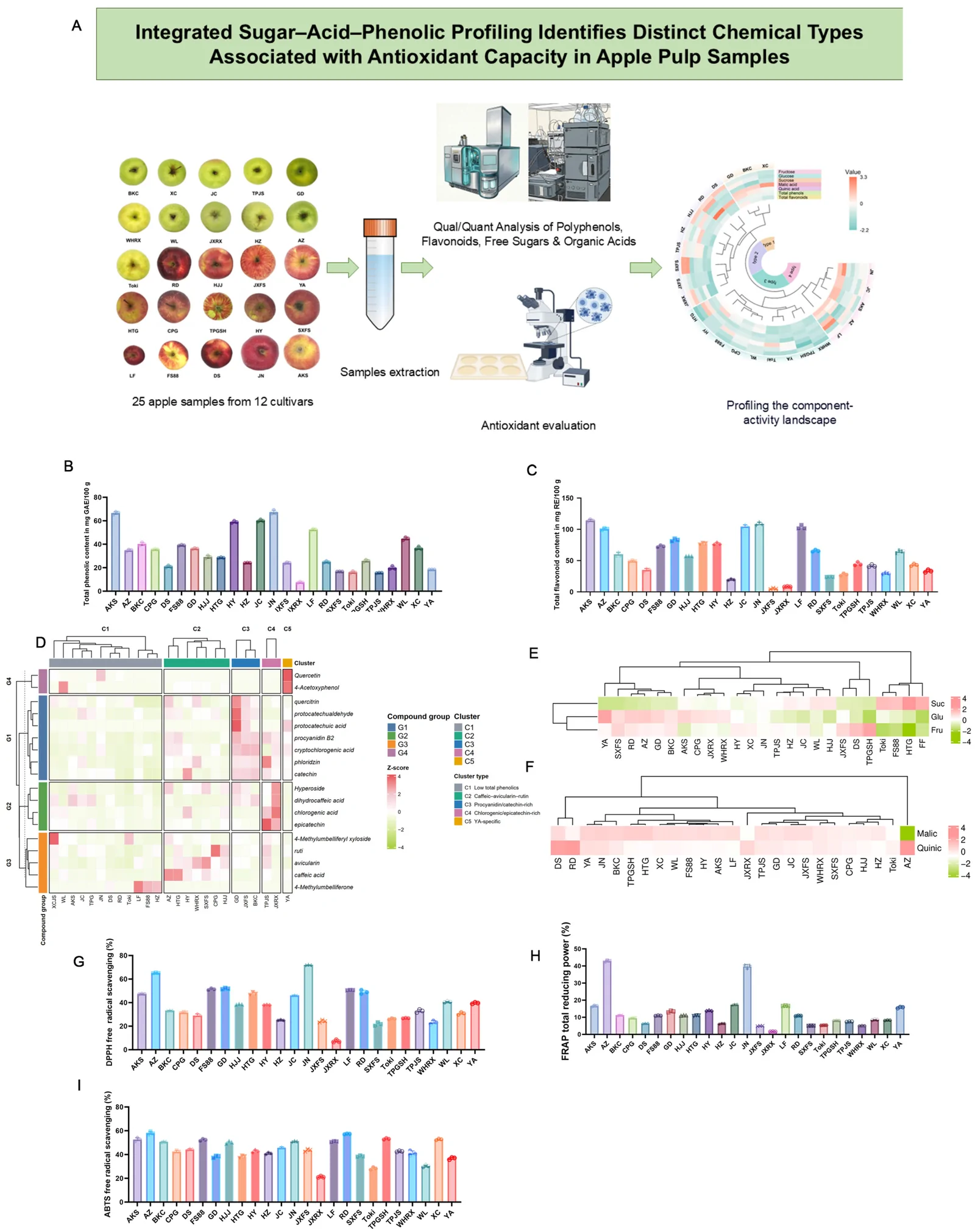
Chemical composition and in vitro antioxidant activity of 25 apple pulp samples from 12 cultivars. (**A**) Apple materials and experimental workflow. (**B**,**C**) Total phenolic and total flavonoid contents, respectively. (**D**) Heatmap of 16 targeted phenolic and flavonoid compounds. (**E**) Heatmap of fructose, glucose, and sucrose contents. (**F**) Heatmap of malic and quinic acid contents. (**G**–**I**) DPPH radical-scavenging activity, FRAP reducing power, and ABTS radical-scavenging activity, respectively. Chemical values are presented as the means of five biological replicates per sample (*n* = 5), with one fruit used for each replicate. Error bars indicate standard deviations. GAE, gallic acid equivalents; RE, rutin equivalents; FW, fresh weight.

The apple pulp samples and experimental workflow are shown in Figure 1A. As illustrated in Figure 1B,C, marked differences in phenolic, sugar, and organic acid compositions were observed among the 25 apple pulp samples from 12 cultivars. Total phenolic content ranged from 6.40 to 75.50 mg GAE/100 g FW, with the highest and lowest levels detected in JN and JXRX, respectively. Total flavonoid content showed even greater variation, ranging from 5.32 to 114.07 mg RE/100 g FW. AKS had the highest total flavonoid content, whereas JXFS showed the lowest value, indicating substantial sample-dependent variation in flavonoid accumulation.

Non-targeted mass spectrometry putatively annotated 32 phenolic and flavonoid compounds at MSI Level 2, providing a broader overview of phenolic diversity among the tested apple pulp samples, with detailed results shown in Supplementary Material S2 [13]. Based on the non-targeted annotation results and known major apple phenolics, 16 representative phenolic and flavonoid compounds were further quantified using authentic standards [6,7]. Chlorogenic acid, epicatechin, and procyanidin B2 were the major quantified phenolic compounds. JN showed the highest chlorogenic acid content, whereas TPGSH contained the highest epicatechin level. Several minor phenolic compounds also showed sample-specific accumulation patterns, suggesting that both major and minor phenolic compounds contributed to the chemical differentiation of the apple pulp samples (Figure 1D). Representative chromatograms and standard curves are provided in Supplementary Materials S2 and S3.

As shown in Figure 1E, fructose, glucose, and sucrose were quantified as the major soluble sugars. Fructose was the predominant sugar in most samples, ranging from 5.02 to 13.70 g/100 g FW. SXFS contained the highest fructose and glucose levels, whereas LF contained the highest sucrose level. Sucrose exhibited the largest coefficient of variation among the three sugars, suggesting greater sample-dependent variation in sucrose accumulation.

As shown in Figure 1F, malic acid and quinic acid were the main detected organic acids, with malic acid being the dominant acid in all samples. Malic acid content ranged from 0.129 to 4.567 g/100 g FW, whereas total quantified acid content ranged from 0.197 to 4.613 g/100 g FW. XC contained the highest malic acid and total acid levels, whereas AZ showed the lowest values. These results indicate substantial variation in sugar and organic acid accumulation among the tested apple pulp samples, providing a chemical basis for subsequent comparisons of chemical profiles and antioxidant capacity.

### 3.2. Chemical Typing Based on Sugar, Acid and Phenolic Profiles

**Figure 2:**
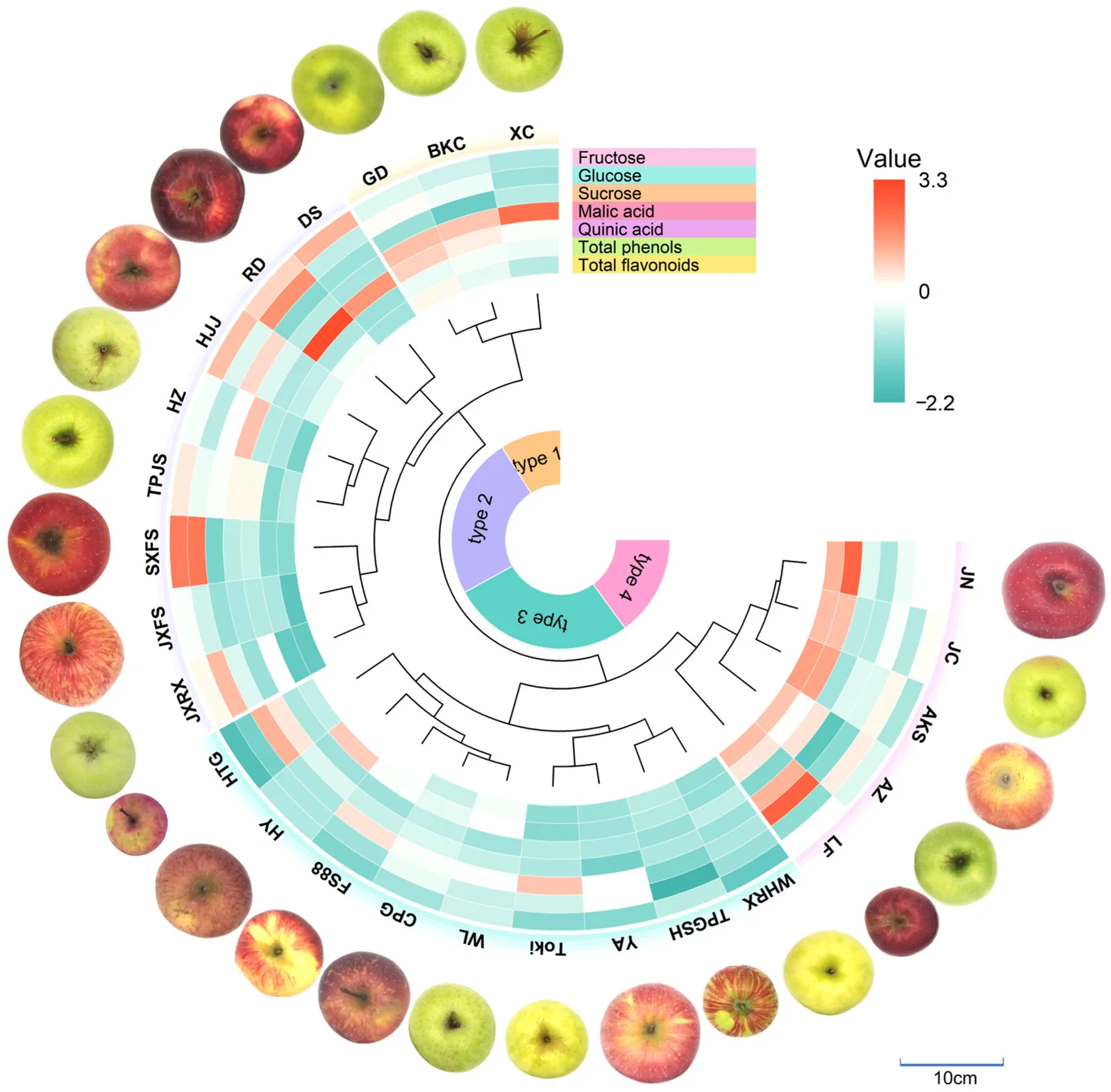
Circular hierarchical clustering heatmap of seven non-redundant chemical traits in 25 apple pulp samples from 12 cultivars. The heatmap was constructed using the mean contents of fructose, glucose, sucrose, malic acid, quinic acid, total phenols, and total flavonoids obtained from five biological replicates. Data for each trait were Z-score standardized across samples, with orange indicating values above the overall mean, white indicating values close to the mean, and turquoise indicating values below the mean. Hierarchical clustering was performed using Euclidean distance and Ward.D2 linkage, and the dendrogram was divided into four sample-level chemical types. The inner colored sectors and outer shaded bands indicate the corresponding type assignments. Type numbers are arbitrary cluster identifiers and do not represent a ranking of sample quality. Representative photographs of the intact fruits are arranged around the heatmap in the corresponding sample order for morphological reference, whereas all chemical measurements were performed using apple pulp. Sample abbreviations and detailed origin information are provided in Table S1.

Hierarchical clustering based on seven non-redundant chemical traits revealed distinct compositional patterns among the 25 apple pulp samples (Figure 2). After Z-score standardization, the samples were divided into four chemical types. Type 1 comprised GD, BKC, and XC and was characterized primarily by relatively high organic acid levels, particularly the marked accumulation of malic acid in XC. Type 2 included DS, RD, HJJ, HZ, TPJS, SXFS, JXFS, and JXRX and generally exhibited a sugar-enriched profile accompanied by comparatively low phenolic-related values. Type 3 consisted of HTG, HY, FS88, CPG, WL, Toki, YA, TPGSH, and WHRX and predominantly showed intermediate or relatively low accumulation across the measured traits. Type 4 included JN, JC, AKS, AZ, and LF and was distinguished mainly by elevated total phenol and/or total flavonoid contents.

The clustering results demonstrate substantial sample-level heterogeneity in the sugar, organic acid, and phenolic composition of apple pulp. Removal of total sugars from the clustering variables avoided redundant weighting of fructose, glucose, and sucrose, while retaining the major chemical patterns. The resulting four chemical types were subsequently used to examine associations between apple pulp composition and antioxidant capacity.

### 3.3. Antioxidant Capacity Across Chemical Types and Its Associations with Chemical Traits

**Figure 3:**
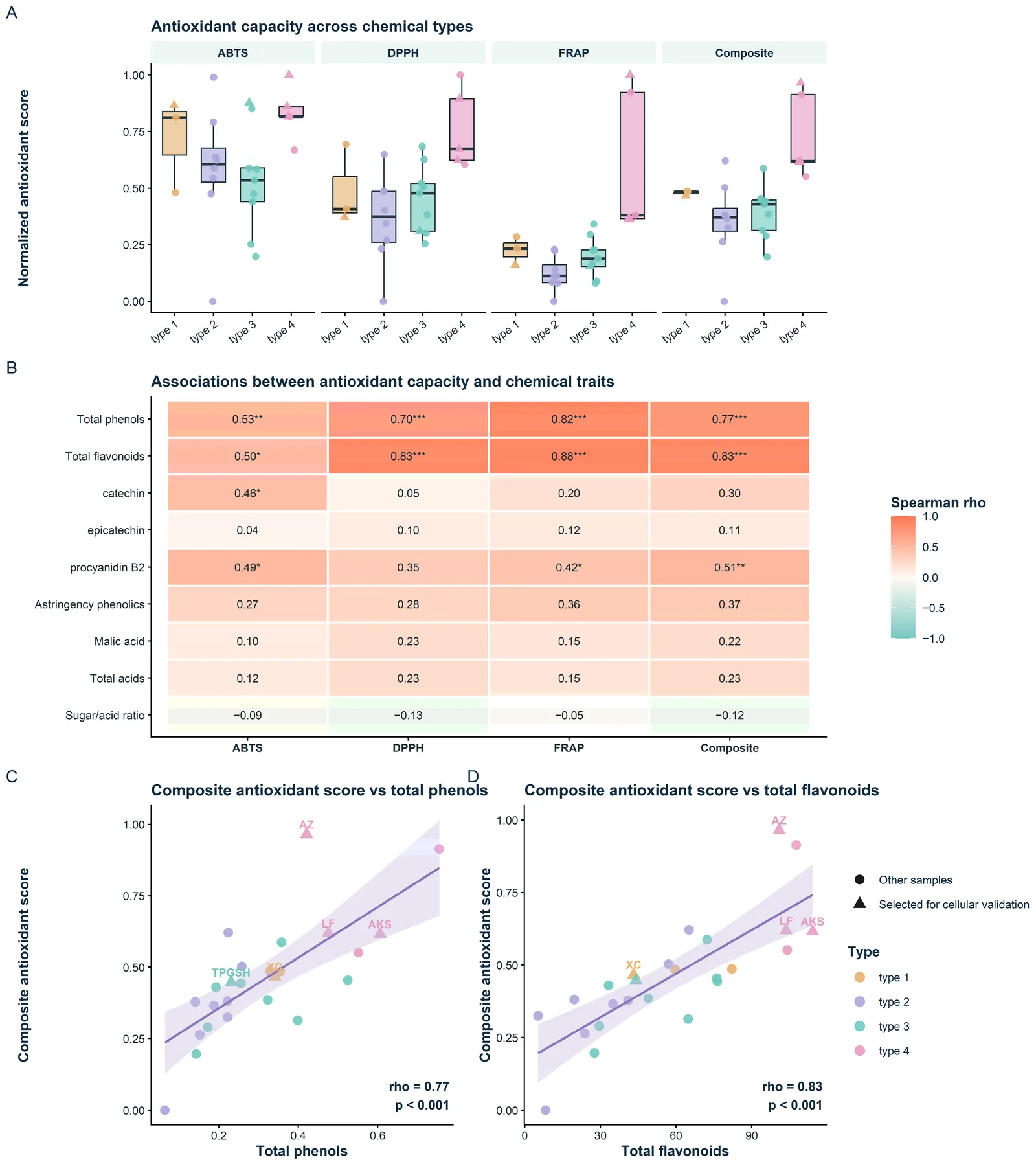
Antioxidant capacity and its associations with chemical traits in 25 apple pulp samples from 12 cultivars. (**A**) Normalized ABTS, DPPH, FRAP, and composite antioxidant scores among the four chemical types. The composite score was calculated as the arithmetic mean of the normalized ABTS, DPPH, and FRAP values. Boxes represent the interquartile range, horizontal lines indicate medians, whiskers extend to 1.5 times the interquartile range, and points represent individual samples. (**B**) Spearman correlations between antioxidant indices and selected chemical traits. Numbers indicate Spearman’s rho; *, **, and *** denote *p* < 0.05, *p* < 0.01, and *p* < 0.001, respectively. (**C**,**D**) Associations of the composite antioxidant score with total phenol (**C**) and total flavonoid (**D**) contents. Lines indicate linear regression fits, and shaded regions represent 95% confidence intervals. Colors indicate chemical types; triangles indicate the five samples selected for cellular validation, and circles indicate the remaining samples.

Antioxidant capacity differed among the four chemical types (Figure 3A). Type 4 showed the highest mean ABTS, DPPH, FRAP, and composite antioxidant scores, whereas types 2 and 3 generally showed lower antioxidant values. Significant differences among chemical types were detected for DPPH (*p* = 0.034), FRAP (*p* = 0.002), and the composite antioxidant score (*p* = 0.009), whereas the difference in ABTS activity was not statistically significant (*p* = 0.093; Kruskal–Wallis tests). These results indicate that the chemical classification based on seven non-redundant traits was associated with differences in antioxidant profiles, although considerable variation remained within individual types.

Spearman correlation analysis showed that antioxidant capacity was primarily associated with phenolic-related chemical traits (Figure 3B). Total phenol content was positively correlated with ABTS (ρ = 0.53, *p* < 0.01), DPPH (ρ = 0.70, *p* < 0.001), FRAP (ρ = 0.82, *p* < 0.001), and the composite antioxidant score (ρ = 0.77, *p* < 0.001). Total flavonoid content showed similar associations with ABTS (ρ = 0.50, *p* < 0.05), DPPH (ρ = 0.83, *p* < 0.001), FRAP (ρ = 0.88, *p* < 0.001), and the composite score (ρ = 0.83, *p* < 0.001). Procyanidin B2 showed weaker and assay-dependent correlations, reaching statistical significance for ABTS, FRAP, and the composite score. By contrast, malic acid, total acids, and sugar/acid ratio showed weak and non-significant associations with the antioxidant indices. These findings suggest that antioxidant variation was more closely associated with phenolic and flavonoid abundance than with sugar–acid composition.

At the individual-sample level, the composite antioxidant score was positively associated with total phenols (ρ = 0.77, *p* < 0.001; Figure 3C) and total flavonoids (ρ = 0.83, *p* < 0.001; Figure 3D). Among the five samples selected for cellular validation, AZ showed the highest composite antioxidant score, while LF and AKS also exhibited relatively high values. TPGSH and XC showed intermediate antioxidant capacities despite belonging to different chemical types. Overall, these results indicate that phenolic-related chemical characteristics were important correlates of in vitro antioxidant capacity in the investigated apple pulp samples, whereas sugar–acid traits showed limited associations.

### 3.4. Selection of Representative Samples and Cytotoxicity Assessment

Based on their chemical profiles and antioxidant results, five representative samples, AZ, XC, LF, TPGSH and AKS, were selected for cellular antioxidant validation. AZ and AKS represented phenolic-rich samples with relatively high in vitro antioxidant capacity, TPGSH represented a sample with exceptionally high epicatechin accumulation, and XC and LF represented high-acid or moderate-antioxidant samples.

**Figure 4:**
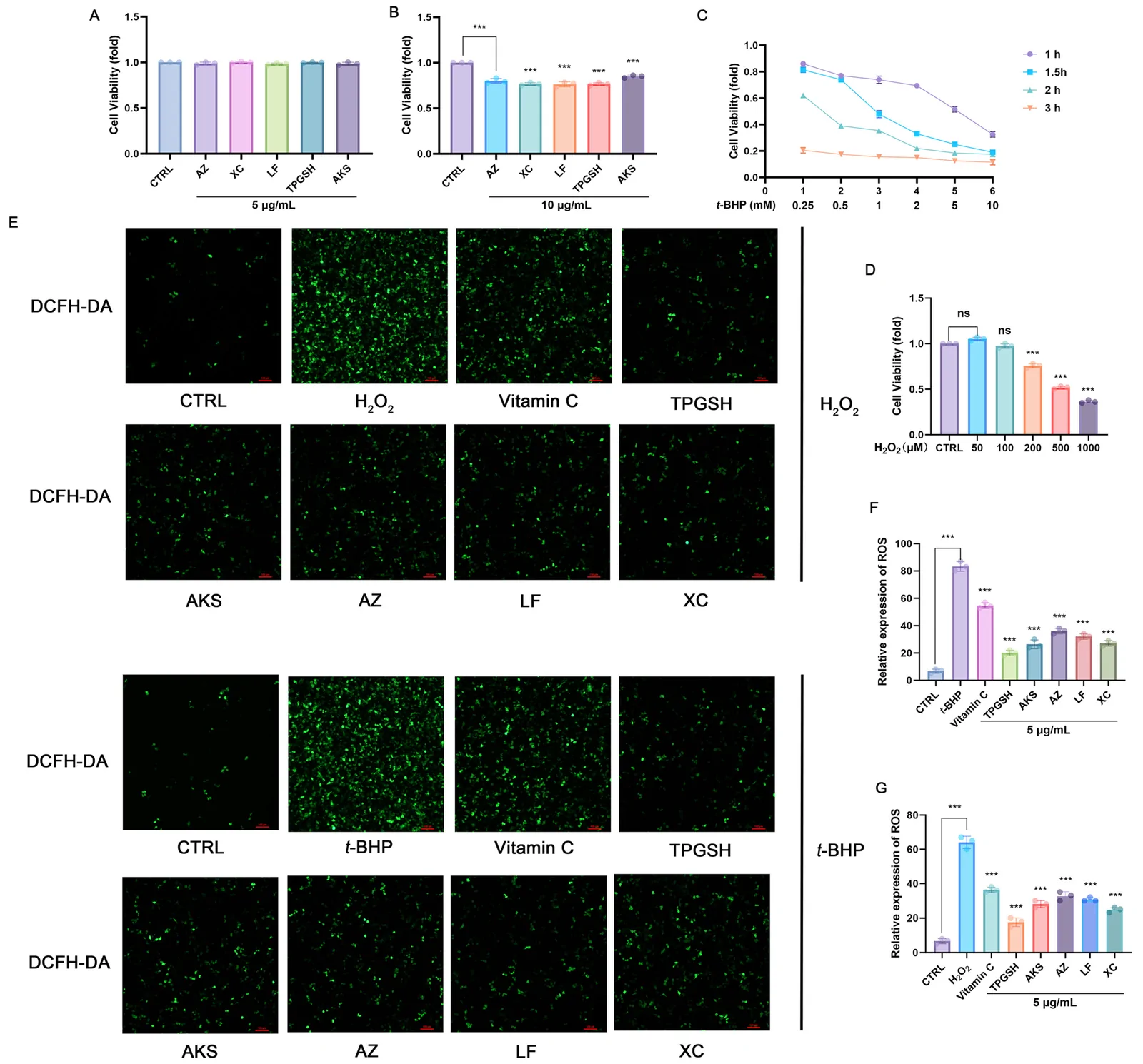
Cytotoxicity assessment and establishment of oxidative stress models in HepG2 cells. (**A**,**B**) Effects of apple pulp polyphenol extracts at 5 and 10 μg/mL, respectively, on cell viability. (**C**) Effects of t-BHP concentration and exposure duration on cell viability. (**D**) Effects of H_2_O_2_ concentration after 1 h of exposure. (**E**) Representative DCFH-DA fluorescence images of intracellular ROS under H_2_O_2_- and t-BHP-induced oxidative stress, following pretreatment with vitamin C or apple polyphenol extracts. (**F**,**G**) Quantification of ROS fluorescence under the H_2_O_2_ and t-BHP models, respectively. Data are presented as mean ± SD (*n* = 3). *** *p* < 0.001, compared with the Model group.

Before cellular antioxidant experiments, the cytotoxicity of apple polyphenol extracts was evaluated using the CCK-8 assay. As shown in Figure 4A,B, 5 μg/mL polyphenol extracts maintained HepG2 cell viability above 95% of the control level (non-significant difference), whereas 10 μg/mL significantly reduced cell viability. Given that the absolute decrease in viability at 5 μg/mL was minimal, this concentration was selected for subsequent experiments to ensure that the observed effects were attributable to antioxidant protection, rather than cytotoxicity. We acknowledge that the limited sample size (*n* = 3) constrains statistical power, and future studies with larger sample sizes are warranted to confirm the safety profile of these concentrations.

### 3.5. Establishment of HepG2 Oxidative Stress Models

As shown in Figure 4C,D, HepG2 cells were exposed to different concentrations of *t*-BHP and H_2_O_2_ to establish suitable cellular oxidative stress conditions. The treatment conditions were selected based on a cell viability of approximately 50% relative to the untreated control. Treatment with 1 mM *t*-BHP for 1.5 h and 500 μM H_2_O_2_ for 1 h produced appropriate levels of oxidative injury and were, therefore, used in the subsequent protection assays.

### 3.6. Cellular Antioxidant Effects of Apple Polyphenol Extracts

As shown in Figure 4E–G, intracellular ROS levels were determined using the DCFH-DA fluorescent probe. Compared with the control group, exposure to H_2_O_2_ or *t*-BHP markedly increased intracellular fluorescence intensity, confirming the successful induction of oxidative stress. Pretreatment with apple pulp polyphenol extracts reduced ROS accumulation to different extents. Among the five tested samples, the TPGSH extract showed the strongest ROS-suppressing effect. This effect may be associated with its high epicatechin content and distinctive phenolic composition, although the specific compounds responsible were not directly established in this study.

**Figure 5:**
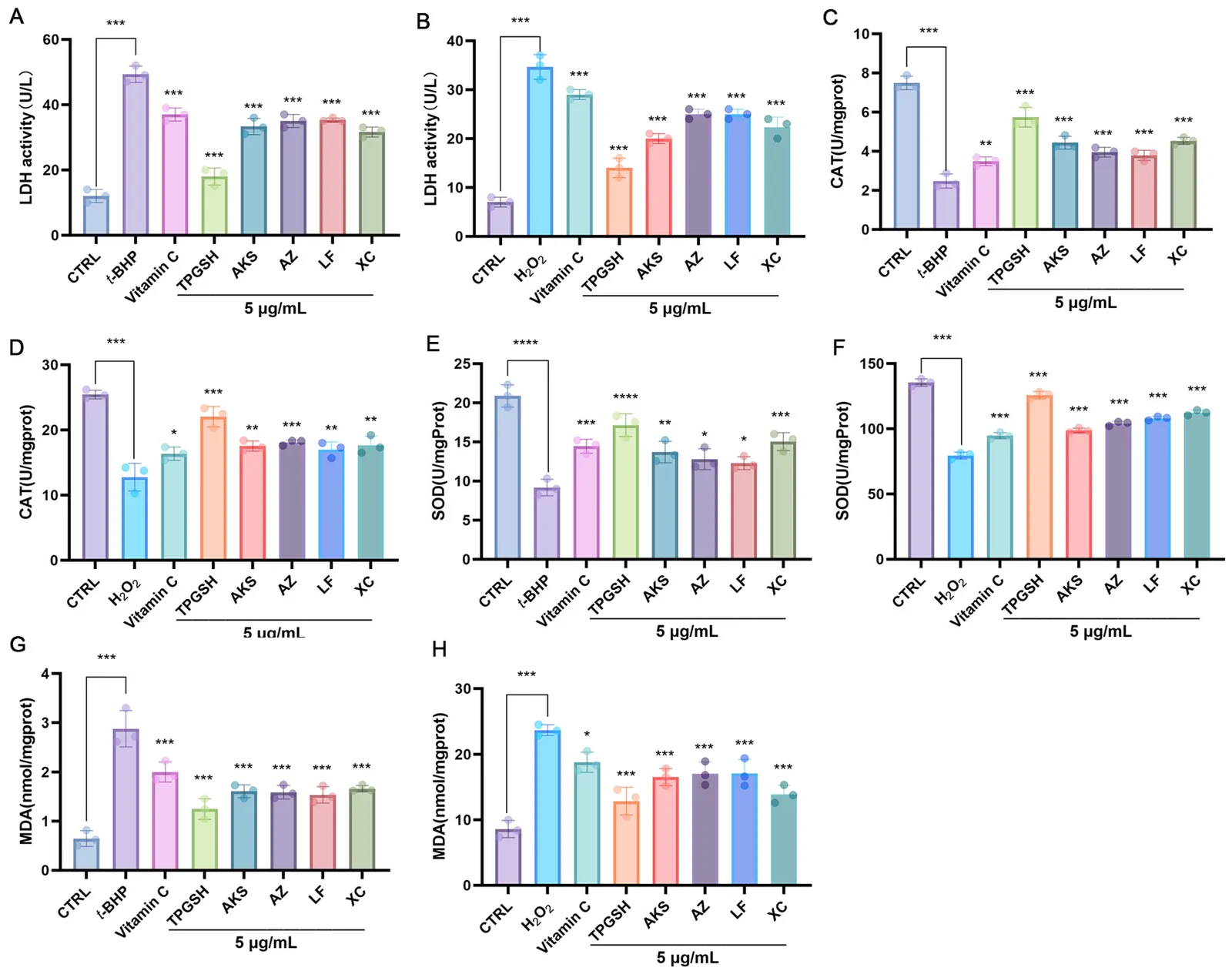
Effects of apple pulp polyphenol extracts on oxidative injury and antioxidant defense in HepG2 cells. LDH release (**A**,**B**), CAT activity (**C**,**D**), SOD activity (**E**,**F**), and MDA content (**G**,**H**) were determined under H_2_O_2_-induced (**A**,**C**,**E**,**G**) and t-BHP-induced (**B**,**D**,**F**,**H**) oxidative stress. Cells were pretreated with vitamin C or polyphenol extracts from the five selected apple pulp samples. Data are presented as mean ± SD (*n* = 3). * *p* < 0.05, ** *p* < 0.01, *** *p* < 0.001, compared with the Model group.

Additional cellular injury-related indicators further supported the protective effects of the apple pulp polyphenol extracts. Oxidative stress treatment increased LDH release and MDA content while decreasing CAT and SOD activities, indicating membrane damage, enhanced lipid peroxidation, and impaired antioxidant defense. As shown in Figure 5, pretreatment with the apple pulp polyphenol extracts reduced LDH release and MDA accumulation and partially restored CAT and SOD activities. The TPGSH extract showed the most pronounced protective effect, followed by the AZ and AKS extracts.

Overall, the cellular experiments demonstrated that apple pulp polyphenol extracts attenuated H_2_O_2_- and t-BHP-induced oxidative stress in HepG2 cells. The stronger cellular protection observed for TPGSH, despite differences between cellular and cell-free antioxidant rankings, suggests that specific phenolic constituents and cellular responses may contribute to cellular antioxidant activity beyond total phenolic content or in vitro antioxidant scores alone.

## 4. Discussion

The present study integrated compositional, cell-free antioxidant, and cellular antioxidant analyses of 25 apple pulp samples from 12 cultivars. Hierarchical clustering based on seven non-redundant chemical traits identified an acid-rich type 1, a sugar-enriched/low-phenolic type 2, an intermediate or low-accumulation type 3, and a phenolic-rich type 4. Total sugars was excluded because it was derived from fructose, glucose, and sucrose, which could have over-weighted sugar-related variation. Type 1 was characterized by relatively high malic acid levels, whereas type 2 showed relatively high sugar-related traits and lower phenolic characteristics. These findings demonstrate that apple pulp samples differed substantially in their sugar, organic acid, and phenolic compositions.

Phenolic-related traits showed clear associations with antioxidant capacity. The phenolic-rich type 4, comprising LF, AZ, AKS, JC, and JN, generally exhibited higher total phenol, total flavonoid, and antioxidant values, whereas the sugar-enriched type 2 showed comparatively lower values. Total phenols and total flavonoids were positively associated with ABTS, DPPH, FRAP, and the composite antioxidant score, while malic acid, total acids, and sugar/acid ratio showed weak associations with antioxidant capacity. These results suggest that phenolic and flavonoid abundance contributed more strongly to antioxidant variation than sugar–acid composition. Differences among ABTS, DPPH, and FRAP rankings were expected because these assays reflect somewhat different reaction mechanisms [15]. Therefore, the composite antioxidant score should be regarded as an exploratory summary, rather than an absolute measure of antioxidant capacity.

The HepG2 experiments provided cellular evidence complementary to the cell-free antioxidant assays [16]. Among the five selected samples, AZ, AKS, and LF belonged to phenolic-rich type 4, XC to acid-rich type 1, and TPGSH to intermediate type 3. Their apple pulp polyphenol extracts reduced intracellular ROS accumulation and alleviated H_2_O_2_- and *t*-BHP-induced oxidative injury. TPGSH showed the strongest cellular protection, despite not belonging to the type with the highest mean cell-free antioxidant capacity. Its high epicatechin content suggests that specific flavan-3-ols, together with cellular uptake, stability, metabolism, and endogenous antioxidant responses, may contribute to cellular activity beyond that predicted by total phenolic content alone [10,17,18,19]. However, the contributions of individual compounds were not directly verified in the present study.

Several limitations should be acknowledged. The samples were collected from different commercial orchards or retail sources, and maturity, agronomic management, storage, and postharvest handling could not be fully standardized. The chemical types therefore represent sample-level compositional patterns, rather than universal cultivar classifications. Correlation analyses cannot establish causality; only five representative samples were examined in the cellular experiments; and the findings apply specifically to apple pulp, rather than peel or whole fruit. In addition, the present study focused mainly on phenolic-related antioxidant potential and did not comprehensively evaluate other nutritional components, such as vitamins, minerals, or dietary fiber [17]. Despite these limitations, the integrated composition–antioxidant framework may assist in selecting apple pulp materials with desirable chemical and antioxidant characteristics, and in developing phenolic-rich apple products.

## 5. Conclusions

This study integrated sugar, organic acid, phenolic, and antioxidant analyses to characterize 25 apple pulp samples from 12 cultivars. Hierarchical clustering based on seven non-redundant chemical traits identified four sample-level chemical types with distinct acid-, sugar-, and phenolic-related profiles. Antioxidant capacity was more closely associated with phenolic and flavonoid abundance than with sugar–acid composition. These relationships represent statistical associations, rather than evidence of direct causality. Cellular assays further showed that selected apple pulp polyphenol extracts reduced oxidant-induced ROS accumulation and cellular injury in HepG2 cells, with TPGSH exhibiting particularly strong protection. Overall, the integrated chemical and antioxidant framework provides a basis for evaluating apple pulp materials according to their compositional and antioxidant characteristics. Further studies addressing standardized maturity and postharvest conditions, bioaccessibility, metabolism, and in vivo efficacy are required to validate the broader applicability of these findings.

## Data Availability

The data presented in this study are currently under patent application and cannot be publicly shared at this time. To request access to the raw data, please submit a formal application to the corresponding author, Professor Lin Tang, for review. Upon approval, data will be provided in compliance with relevant patent regulations and access permissions.

## Supplementary Materials

The following supporting information can be downloaded at: https://www.mdpi.com/article/10.3390/foods15183353/s1, Supplementary Material S1—Abbreviations and Origins of Apple Varieties, Chromatographic conditions and reagent catalog number; Supplementary Material S2—Untargeted Mass Spectrometry Results and Standard Curves Presented in This Paper; Supplementary Material S3—Chromatographic Comparison Charts.
