# Supplementary material for: Integrated Sugar–Acid–Phenolic Profiling Identifies Distinct Chemical Types Associated with Antioxidant Capacity in Apple Pulp Samples
Source: Foods. 2026 Sep 21;15(18):3353. doi: 10.3390/foods15183353 (PMC13606904; doi:10.3390/foods15183353)
Supplement: Supplementary file 1 [file foods-15-03353-s001.zip › Supplementary Material S3 - Chromatographic Comparison Charts.pdf]

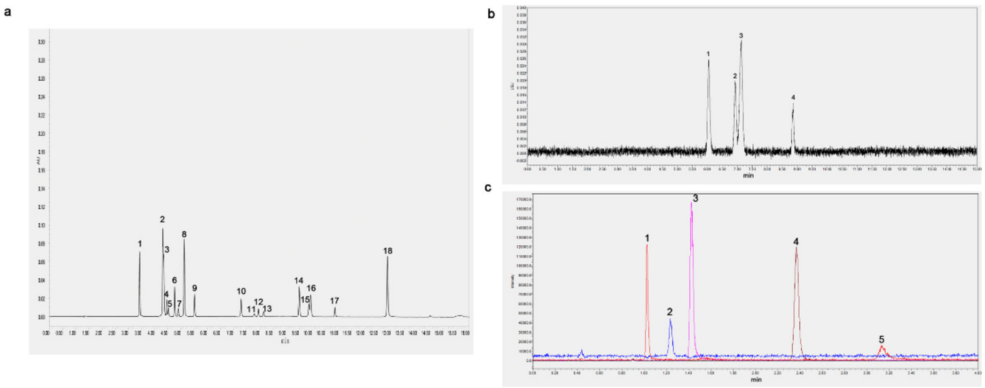

a:UPLC Chromatogram of Phenolic Standard Mixtures; b: UPLC-ELSD chromatogram of free sugar standards 1 Glucose 2 Mannitol 3 Fructose 4 Sucrose; c: LC-MS Chromatograms of Certified Organic Acid Reference 1 Quinic acid 2 Vitamin C 3 Malic acid 4 Succinic acid 5 Citric Acid.

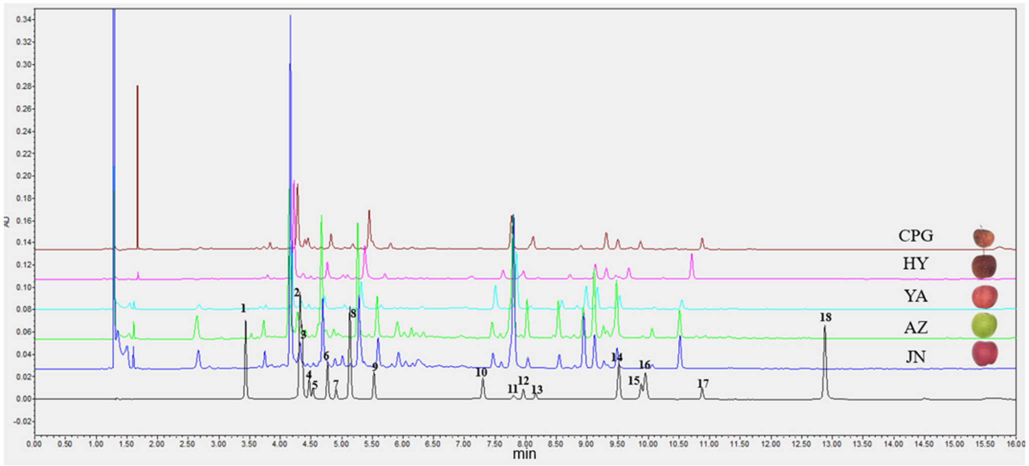

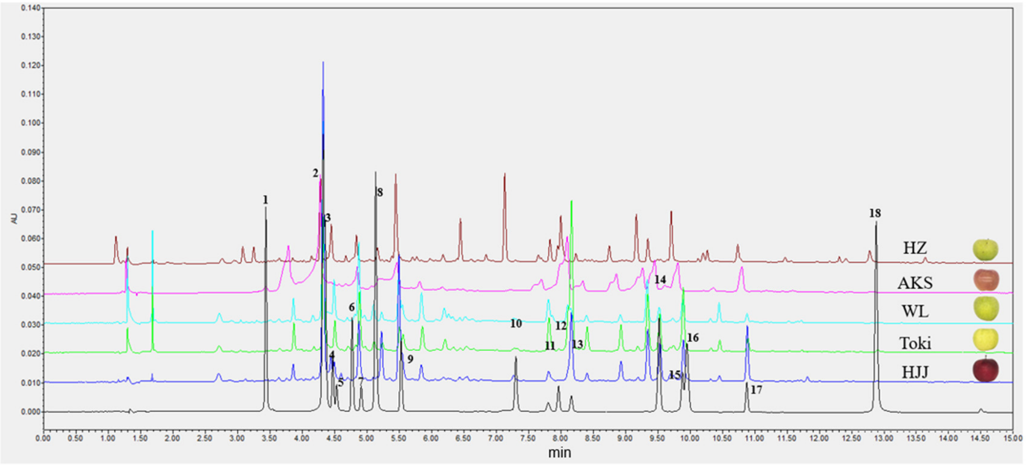

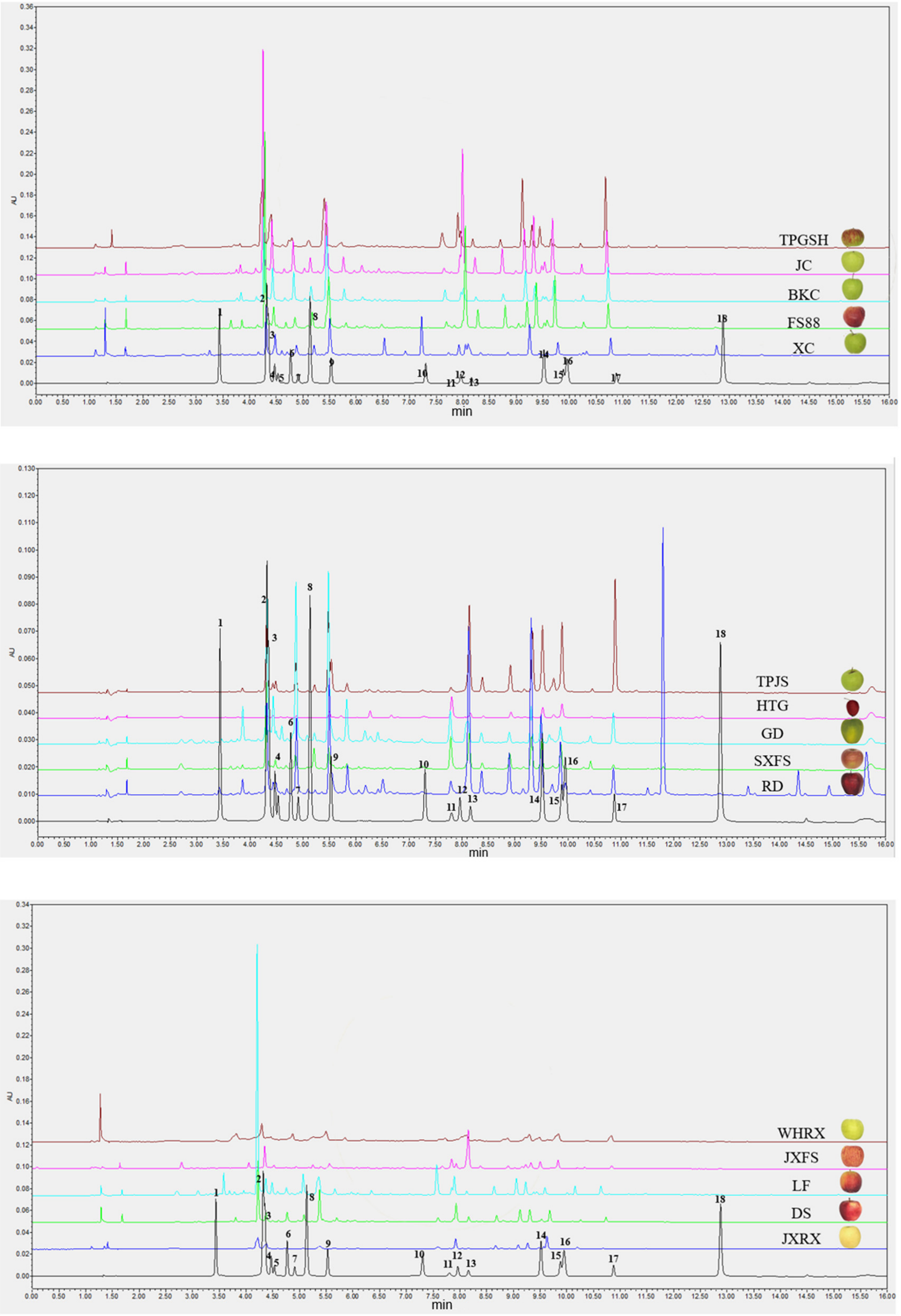

Chromatograms of 25 apple polyphenol varieties and the control sample.

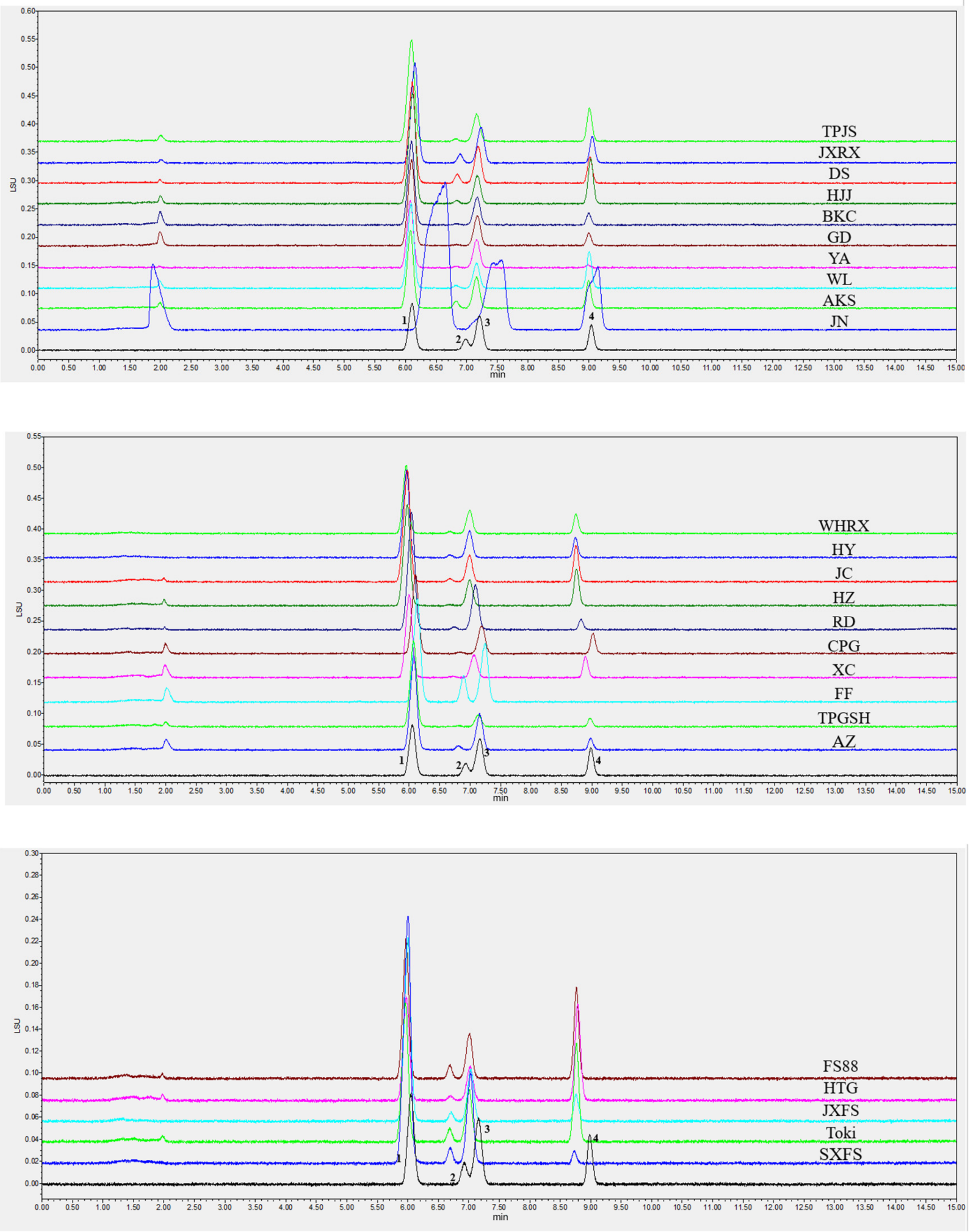

UPLC-ELSD chromatograms of free sugars in five apple cultivars

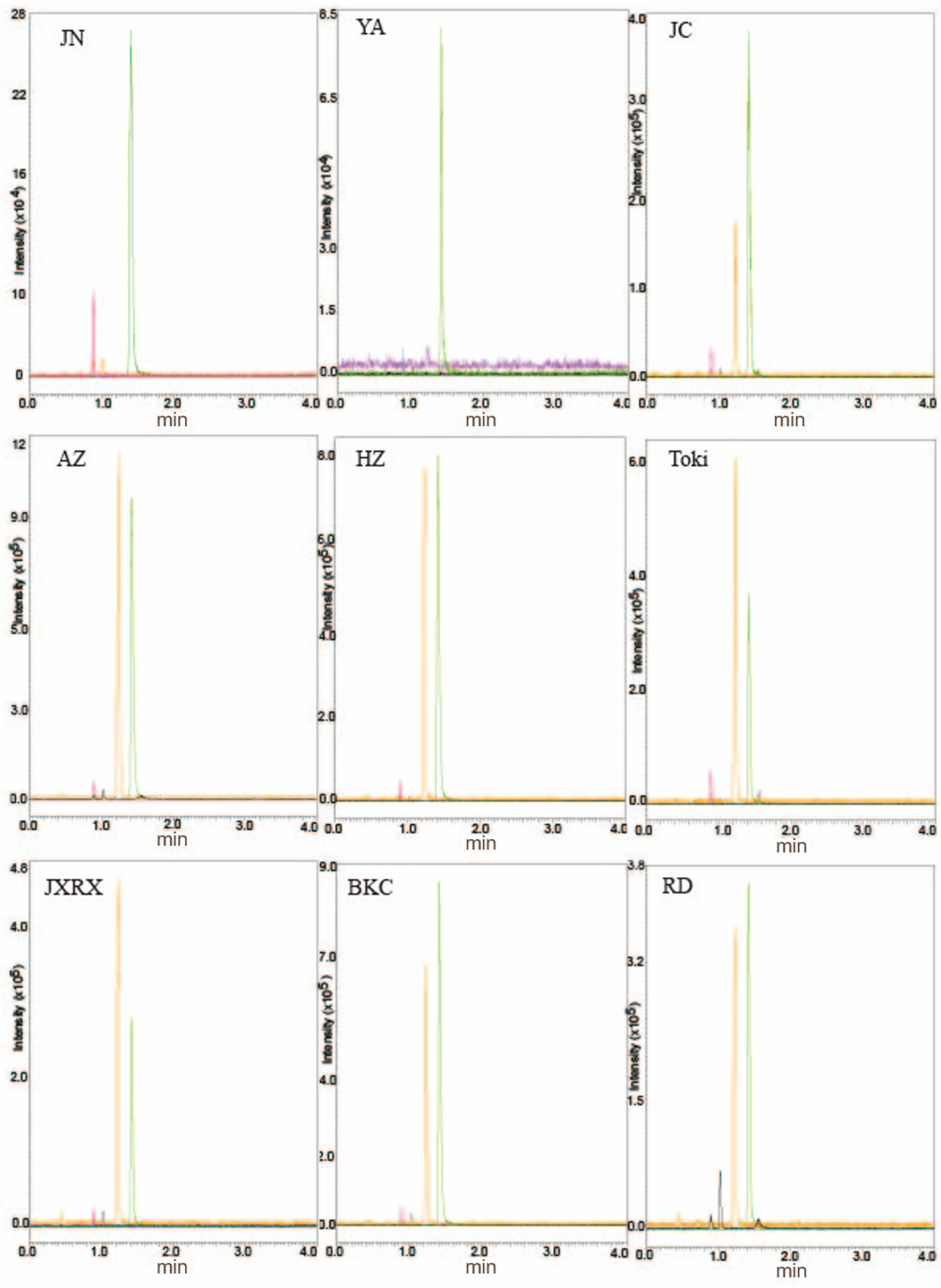

JN
YA
JC
AZ
HZ
Toki
JXRX
BKC
RD
Intensity (x10⁴)
Intensity (x10⁵)
min

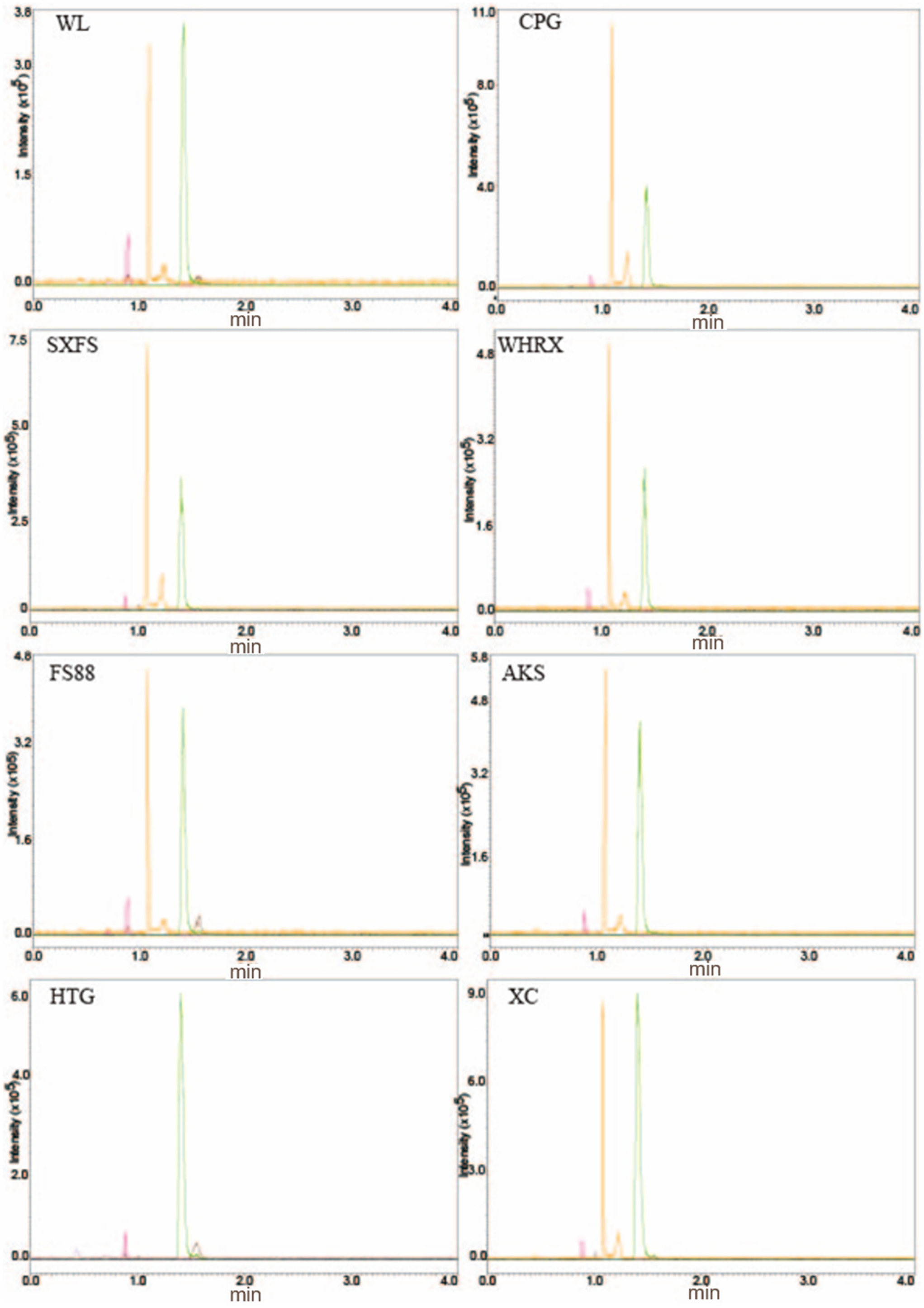
WL
CPG
SXFS
WHRX
FS88
AKS
HTG
XC
Intensity (x10^5)
min

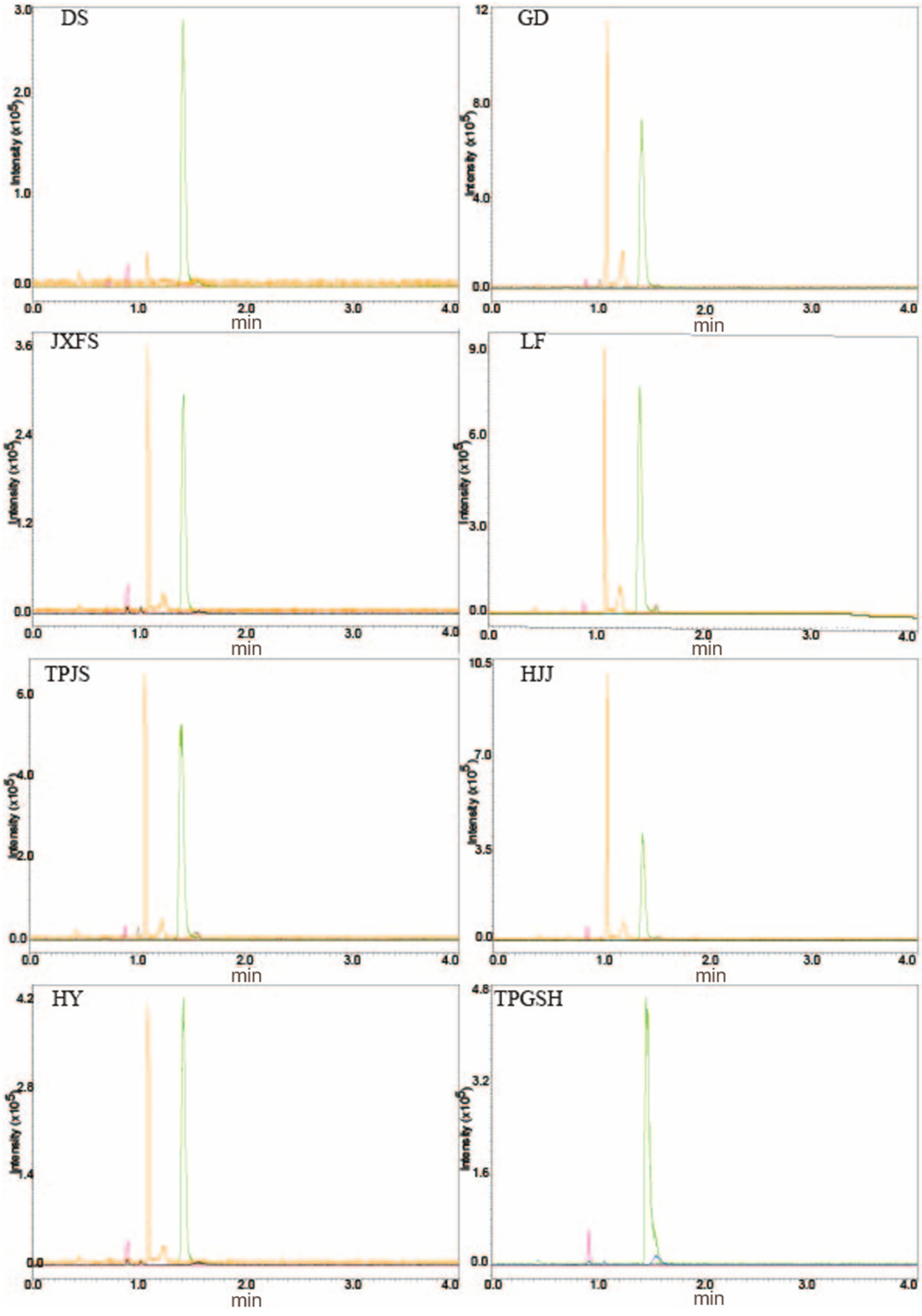

LC-MS Chromatographic Profiles of Characteristic Organic Acids in Nine Apple Varieties (DS, GD, JXFS, LF, TPJS, HJJ, HY, TPGSH) Note: Purple - quinic acid; yellow - ascorbic acid; green - malic acid
